# SNP Variation of *RELN* Gene and Schizophrenia in a Chinese Population: A Hospital-Based Case–Control Study

**DOI:** 10.3389/fgene.2019.00175

**Published:** 2019-03-05

**Authors:** Xia Luo, Si Chen, Li Xue, Jian-Huan Chen, Yan-Wei Shi, Hu Zhao

**Affiliations:** ^1^Department of Psychiatry, Shenzhen Kangning Hospital, Shenzhen Mental Health Center, Shenzhen, China; ^2^Department of Psychiatry, Shantou University Medical College, Shantou, China; ^3^Faculty of Forensic Medicine, Zhongshan School of Medicine, Sun Yat-sen University, Guangzhou, China; ^4^Institute of Biomedical and Pharmaceutical Sciences, Guangdong University of Technology, Guangzhou, China; ^5^Laboratory of Genomic and Precision Medicine, Wuxi School of Medicine, Jiangnan University, Wuxi, China; ^6^Guangdong Province Translational Forensic Medicine Engineering Technology Research Center, Sun Yat-sen University, Guangzhou, China

**Keywords:** *RELN*, schizophrenia, SNP, association analysis, quantitative trait loci analyses, psychiatric symptoms, bioinformatics tools

## Abstract

**Aims:** We aimed to explore whether *RELN* contributes to the vulnerability and severity of clinical symptoms of schizophrenia (SZ) in a Chinese population.

**Methods:** The following were conducted in an adult Han Chinese population from southern China: case–control association analyses of 30 representative single nucleotide polymorphisms (SNPs) that were screened according to specific programs based on bioinformatics tools and former research and quantitative trait locus analyses with SNPs and psychiatric symptoms evaluated with the positive and negative symptoms scale.

**Results:** A 4-SNP haplotype consisting of rs362814, rs39339, rs540058, and rs661575 was found to be significantly associated with SZ even after Bonferroni correction (χ^2^ = 29.024, *p* = 6.42E-04, *p*_Bonf_ = 0.017), and the T-C-T-C haplotype was a protective factor for SZ (OR = 0.050, 95% CI = 0.004–0.705). Moreover, the 4-SNP haplotype showed a significant association with G16 (active social avoidance) after false discovery rate correction (χ^2^ = 28.620, *p* = 1.697E-04, *p*_FDR_ = 0.025). In addition, P7 (hostility) was related to the haplotype comprising rs2229864, rs2535764, and rs262355 (χ^2^ = 31.424, *p* = 2.103E-05, *p*_adjustment_ = 0.019) in quantitative trait loci analyses.

**Conclusion:** Overall, this study showed several positive associations between *RELN* and SZ, as well as psychiatric symptoms, which not only supports the proposition that *RELN* is a susceptibility gene for SZ but also provides information on a genotype-phenotype correlation for SZ in a Chinese population.

## Introduction

Schizophrenia (SZ, Online Mendelian Inheritance in Man [OMIM] 181500) is a common and serious psychiatric disorder with a lifetime prevalence estimate of 4.0 (1.6–12.1) per 1,000 individuals (Esan et al., 2012). SZ has been reported to be a predominantly genetic disorder, in which heritability is estimated to be 80% (Lichtenstein et al., 2009).

The human *RELN* gene (OMIM 600514) maps to chromosome 7q22 and encodes reelin, a large secreted glycoprotein, that is thought to be critical for cell positioning and neuronal migration by controlling cell-cell interactions during brain development (Hartfuss et al., 2003; Huang and D’Arcangelo, 2008; Niu et al., 2008). Recently, growing evidence has shown that the reelin protein might also be associated with neurotransmission, memory formation and synaptic plasticity (Herz and Chen, 2006), which have been demonstrated to also be damaged in SZ patients (Falkai et al., 2015). Decrease trends in reelin expression in patients with SZ have been found in brain and blood tissues (Impagnatiello et al., 1998; Guidotti et al., 2000; Nabil Fikri et al., 2017). Hence, low level or dysfunctional of reelin protein may cause deficits in neuronal development and cognitive function in adults and may play pathogenic roles in neuropsychiatric illnesses, such as SZ. This proposition was further supported by anatomical and *in vivo* studies that included the reeler mice and the heterozygous reeler mouse (HRM) model (Hill et al., 2006; Qiu et al., 2006; Tueting et al., 2006). The role of *RELN* as a potential risk for SZ has also been suggested by genetic association analysis, especially in GWASs (Shifman et al., 2008; Li et al., 2011, 2013; Ovadia and Shifman, 2011; Zhou et al., 2016). Over the past decade, dozens of SNPs in *RELN* gene loci have been reported to be associated with the onset and/or severity of clinical symptoms of SZ (Kahler et al., 2008; Shifman et al., 2008; Wedenoja et al., 2008, 2010; Need et al., 2009; Ben-David et al., 2010; Liu et al., 2010; Kuang et al., 2011; Li et al., 2013). However, the results remain controversial (Tost and Weinberger, 2011; Li et al., 2013; Bocharova et al., 2017).

In this study, we assumed that RELN may be related to the onset of SZ and the severity of some clinical symptoms. A case–control study had been performed in the Han Chinese population from southern China. To thoroughly understand the genetic basis of mental symptoms in SZ rather than only focus on verifying previous positive results and searching for new susceptible SNPs in *RELN*, we specifically performed quantitative trait locus (QTL) analyses in addition to qualitative association studies. Due to the high clinical and genetic heterogeneity of SZ, different clinical subtypes of SZ may be related to different genetic bases, we specifically collected patients with paranoid or undifferentiated SZ who experienced first onset or recurrence after drug withdrawal at least 1 month to improve the consistency of research subjects and to minimize the influence of drug on the scores of PANSS.

## Materials and Methods

### Subjects

The patient sample consisted of 102 unrelated SZ patients (46 females and 56 males; mean age, 32.42 ± 10.08 years) recruited from the Third People’s Hospital of Zhongshan City during 2012.11–2013.06. Patients were diagnosed by at least two psychiatrists according to the criteria of the Diagnostic and Statistical Manual of Mental Disorders (Fourth Edition, DSM-IV). Detailed information on clinical features, such as the onset time, symptoms and family history of mental illness, were obtained according to DIGS and FIGS. The PANSS was used to evaluate the severity of psychosis symptoms of SZ patients. It is one of the most common instruments applied to evaluate the severity of clinical symptoms in the world (Kay et al., 1987). PANSS comprises 33 items, including 30 psychopathological items which are usually divided into the positive subscale (7 items), the negative subscale (7 items) and the general psychopathology subscale (16 items) and 3 complementary attack risk items. The item level of the 33 items ranges from 1 to 7, with 1 equaling “no symptoms” (Kay et al., 1987). The Chinese version of PANSS, with acceptable validity and reliability, was used to evaluate the severity of psychosis symptoms of SZ patients in this study (Si et al., 2004). Paranoid or undifferentiated SZ patients who were at first onset, having never been treated, or at recurrence, having not taken any antipsychotics for at least 1 month, were enrolled in. Among them, patients with the both-negative type (i.e., the number of items with a score greater than or equal to 4 was less than three on both positive and negative subscales in PANSS) were excluded. All patients with ambiguous diagnoses or accompanied by neurological diseases, organic mental disorders or other symptomatic psychoses, or who had concomitant severe somatic disease, cancer, pregnancy or lactation, an immune or endocrine system disease, or a history of alcoholism or substance abuse were excluded.

A total of 169 healthy controls (75 females and 94 males; mean age 33.27 ± 8.63 years) matched with the patients in sex, age, and birthplace at the same time were randomly recruited from the volunteers who came to perform physical examination in the Affiliated Hospital of Sun Yat-sen University during 2013.06–2013.11. Volunteers were asked to provide detailed information on medical and family psychiatric histories. Subjects were excluded due to any of the following: positive family histories (first-degree relatives) of psychiatric illness; substance abuse; abnormal birth; febrile convulsions; juvenile adoption or residence with a single-parent family; pregnancy or lactation.

All participants were unrelated Han Chinese born and living in the southern China (mostly come from Guangdong province and nearby cities), and all their biological grandparents were Han Chinese ancestry. They provided written informed consent for participation, and the research protocol was approved by the Ethical Committee for Genetic Studies of Shantou University.

### SNP Selection

**FIGURE 1:**
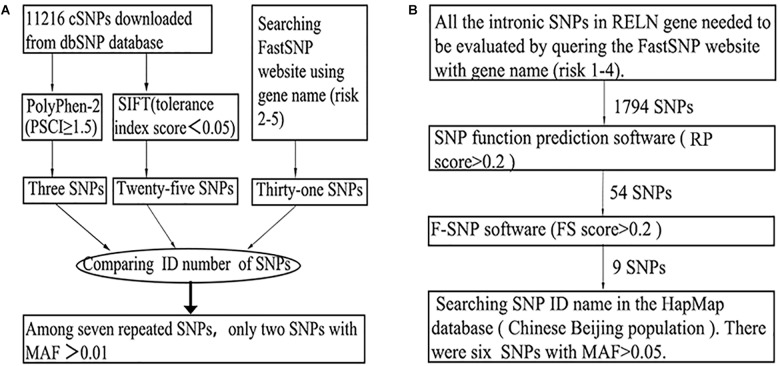
The general screening flows of exonic **(A)** and intronic **(B)** SNPs in the SNP selection phase.

The SNPs were based on previous studies (Shen et al., 2006; Doss et al., 2010; Wu and Jiang, 2013) and/or screened by SNP functional prediction softwares, including SIFT (Kumar et al., 2009), PolyPhen-2 (Adzhubei et al., 2010), and FASTSNP (Yuan et al., 2006). Information on the SNPs was obtained from dbSNP, HapMap and other human genome databases. A SNP would be approved only when its MAF was greater than or equal to 0.01 in the Chinese Beijing population in the HapMap database. The detailed strategies for SNP selection were as follows. (1) “Positive” SNPs that have been reported to be associated with SZ previously were selected once their MAFs were greater than or equal to 0.01 in the Chinese Beijing population in the HapMap database. (2) A two-step method was executed for the selection of exonic SNPs (Figure 1A). First, PolyPhen-2, the SIFT online service and the FASTSNP online service were employed for functional analysis of exonic SNPs. Next, by comparing the outputs of the three software programs, repeated SNPs with MAFs equal to or greater than 0.01 were chosen. (3) For intronic SNPs, a four-step method was conducted using the FASTSNP, SNP functional prediction (Xu et al., 2007), F-SNP online services and HapMap database successively, and SNPs with MAFs greater than or equal to 0.05 were preserved (Figure 1B). (4) By using FASTSNP, UTRscan (Shen et al., 2006), and UCSC Variant Annotation Integrator online software (Meyer et al., 2013), similar functional analyses of exonic SNPs were conducted among UTR and upstream SNPs of *RELN*, but a higher MAF threshold of 0.05 was implemented. Rs7341475, a “positive” SNP thought to be associated with SZ (Shifman et al., 2008; Ben-David et al., 2010) was not included in this study, as there was no significant evidence implying that rs7341475 may associate with SZ in the Han Chinese population (Li et al., 2011, 2013).

### SNP Genotyping

For each participant, 5 ml peripheral venous blood was collected after signing informed consent and before drug used. Genomic DNA was extracted using the phenol-chloroform method within 1–2 weeks and saved in −20°C refrigerator. SNP genotyping was performed with the Sequenom MassARRAY iPLEX Gold platform (Sequenom, San Diego, CA, United States) (Ikeda et al., 2008). Primers were designed using the MassARRAY Assay Design 3.1 software. Two amplification primer systems were built with the balance of capturing as many SNP loci as possible while limiting costs. Genomic fragments containing SNPs were amplified by polymerase chain reaction (PCR) in a total reaction volume of 5 μl, which included 20–50 ng of genomic DNA, in two 384-well plates using the ABI GeneAmp^®^ 9700 384 Dual. Purified and specific genotyping primers were used to amplify target sites. Genotypes were automatically called by MassARRAY Typer 4.0 and verified manually. To ensure the accuracy of genotyping, 32 samples were duplicated for quality control, and no genotyping errors were found. Additionally, each 384-well plate had four blank controls. Any individual whose missing genotypes were greater than 50% was excluded from further statistical analysis.

### Statistical Analysis

Statistical analyses including HWE, the description of MAF, single marker association of allele, the standardized measure of linkage disequilibrium (LD) coefficients (D’), haplotype block and haplotype frequency and association within the block were all assessed using Haploview v4.2 software (version 4.2, Broad Institute of MIT and Harvard, Cambridge, MA, United States) (Gabriel et al., 2002; Barrett et al., 2005). The haplotype frequency was estimated using the expectation maximization (EM) algorithm. The criterion for significances in Haploview v4.2 was set at p smaller than 0.05 for tests of expected HWE and corrected *p*-value after 10000 permutations.

The SPSS 19.0 were used to analyzing the demographic and genotypic frequency distribution of samples using *t*-tests or chi-square tests. Logistic regression analyses of population, sex, and age were used to generate population stratification assignments for all individuals. The criterion for significance in SPSS 19.0 was set at p smaller than 0.05. Genotype and multiple SNPs qualitative traits association tests between patients and controls were performed with Unphased 3.1.7 (Dudbridge, 2008). The overall test of association in Unphased 3.1.7 is a likelihood ratio test. And the odds ratio (OR) and 95% confidence interval (95% CI) were calculated automatically to evaluate the effects of alleles and haplotypes. To further investigate the possibility of complexity in the genotype-phenotype relationship, QTL analyses were also performed with Unphased v3.1.7 using 33 factor scores and 4 scale scores (i.e., the total scale score, the positive scale score, the negative scale score, and the general psychopathology scale score) from PANSS as quantitative traits. In QTL analyses, the addVal (i.e., additive genetic value) instead of OR was calculated automatically to show the estimated additive genetic value for special haplotype. For all multiple tests performed in Unphased 3.1.7, Bonferroni correction and FDR correction were employed to correct *p*-values and minimize the influence of type II errors. The corrected *p*-value threshold of 0.05 was used for significant after Bonferroni correction (*p*_Bonf_ = α × m, α is the desired overall alpha level of 0.05, and m is the number of hypotheses) and FDR correction (the corrected *p*-value was shown as *p*_FDR_) which was performed using R projection Version 3.1.2^1^. A power analysis was performed using the G^∗^Power software for this study (Faul et al., 2009).

## Results

### SNP Selection and Genotyping

**FIGURE 2:**
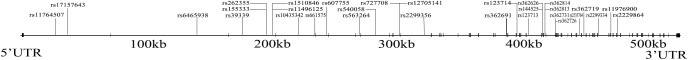
The genomic structure of *RELN* and the physical locations of the 30 tested SNPs. Among them, rs362691 and rs2229864 are located in the exonic areas (vertical bars), and the others are located in the intronic areas (spaces).

Initially, thirty-nine SNPs were screened, including twenty-nine “positive” SNPs reported previously and ten “functional” SNPs, from more than ten thousand *RELN* loci. Screening flows and the number of targeted SNPs in exonic and intronic zones in the *RELN* gene are shown in Figure 1. Six SNPs (i.e., rs362746, rs12705169, rs885995, rs11761011, rs16872603, and rs2237628) were ruled out for poor specificity or missing data, and three SNPs (i.e., rs3025962, rs73714410, and rs7811571) were monomorphic in the samples. Therefore, 30 SNPs in the *RELN* gene were genotyped successfully in 100 SZ patients (45 females and 55 males; mean age, 32.170 ± 10.000 years) and 163 healthy controls (74 females and 89 males; mean age, 33.350 ± 8.585 years). No differences were found between patients and controls in age (*t* = 1.015, *df* = 261, *p* = 0.311) and gender (χ^2^ = 0.004, *df* = 1, *p* = 1.000). The average call rate of all SNPs was 99.5% in total sample. No SNP deviation from HWE was found in the controls (Table 1). Among 30 SNPs, there are 2 exonic SNPs and 28 intronic SNPs. The rough distribution of the 30 SNPs is shown in Figure 2.

**Table 1:** Genotype frequencies, HWE tests, and single-SNP association analyses of 30 RELN SNPs in the SZ and control cases.

dbSNP ID	Allele (D/d)^a^	Case						Control						Genotype (*p*)^e^	Allele (*P*)^e^
		n^b^	HWE (*p*)^c^	Genotype			MAF^d^	n^b^	HWE (*p*)^c^	Genotype			MAF^d^		
				DD	Dd	dd				DD	Dd	dd			
rs11764507	G/A	100	0.068	72	22	6	0.170	161	0.381	106	52	3	0.180	0.062	0.768
rs17157643	T/A	100	1.000	78	21	1	0.115	162	0.734	137	25	0	0.077	0.213	0.145
rs6465938	C/T	100	0.046	40	38	22	0.410	163	0.388	60	83	20	0.377	0.048	0.455
rs39339	A/C	100	0.416	77	20	3	0.130	162	0.714	126	35	1	0.114	0.283	0.589
rs155333	G/A	100	0.123	54	34	12	0.290	162	0.546	78	72	12	0.296	0.201	0.878
rs262355	T/A	99	0.071	55	32	12	0.283	162	0.700	80	70	12	0.290	0.173	0.858
rs1510846	T/C	95	0.834	29	49	17	0.437	162	0.210	50	72	40	0.469	0.408	0.478
rs11496125	C/T	98	1.000	36	47	15	0.393	162	0.447	65	71	26	0.380	0.850	0.764
rs10435342	C/T	100	0.277	85	13	2	0.085	163	0.995	143	19	1	0.064	0.599	0.376
rs661575	C/T	99	0.131	51	34	14	0.308	163	0.072	81	60	22	0.319	0.958	0.794
rs607755	A/G	99	1.000	37	47	15	0.389	160	0.182	47	88	25	0.431	0.429	0.342
rs563264	A/G	100	1.000	95	5	0	0.025	162	0.443	150	11	1	0.040	0.386	0.356
rs540058	T/C	98	1.000	93	5	0	0.026	161	0.446	149	11	1	0.040	0.404	0.370
rs727708	A/G	100	0.943	30	51	19	0.445	160	1.000	36	80	44	0.475	0.184	0.076
rs12705141	A/T	100	0.664	74	23	3	0.145	161	0.747	110	45	6	0.177	0.528	0.338
rs2299356	A/G	100	0.406	34	53	13	0.395	161	0.880	47	82	32	0.453	0.344	0.190
rs362691^f^	G/C	100	1.000	84	15	1	0.085	163	1.000	121	39	3	0.138	0.153	0.067
rs123714	C/T	100	0.763	52	40	10	0.300	163	0.614	91	64	8	0.245	0.247	0.169
rs123713	C/T	100	0.763	50	40	10	0.300	161	0.899	90	62	9	0.248	0.339	0.196
rs144525	A/G	99	0.536	69	29	1	0.157	161	0.380	101	56	4	0.199	0.448	0.227
rs362626	C/A	100	1.000	40	46	14	0.370	161	0.307	61	82	18	0.366	0.628	0.935
rs362814	A/T	99	0.613	41	43	15	0.369	161	0.497	62	80	19	0.366	0.521	0.959
rs362813	T/C	100	0.961	26	49	25	0.495	163	0.694	47	78	38	0.472	0.857	0.614
rs362731	T/C	100	1.000	26	50	24	0.490	162	0.759	47	78	37	0.469	0.856	0.642
rs362726	T/C	98	0.879	24	51	23	0.495	163	1.000	56	79	28	0.414	0.189	0.072
rs2535764	C/T	99	0.066	68	24	7	0.192	163	0.352	106	48	9	0.202	0.647	0.769
rs362719	C/A	100	0.926	36	47	17	0.405	159	0.131	74	62	23	0.340	0.258	0.132
rs2299334	G/A	100	0.680	66	32	2	0.180	163	1.000	104	52	7	0.202	0.612	0.527
rs11976900	A/G	99	0.486	57	34	8	0.253	161	0.139	92	54	15	0.261	0.951	0.833
rs2229864^f^	C/T	100	1.000	68	29	3	0.175	161	0.214	104	47	10	0.208	0.545	0.354

*^a^Major/minor allele, major, and minor alleles are denoted by D and d, respectively. ^b^Numbers of samples that are successfully genotyped in patients and controls, respectively. ^c^p-Values of the Hardy-Weinberg equilibrium (HWE) tests from Haploview v4.2. ^d^The minor allele frequency of 30 selected SNPs in this study. ^e^Uncorrected p-values of allelic and genotypic association analysis in this study. ^f^Two exonic SNPs selected in this report.*

### Single-SNP Association Analyses

Detailed information regarding the allelic and genotypic frequencies of the 30 SNPs from patients and controls was obtained (Table 1). No single polymorphism was found to be significantly associated with SZ neither in the total sample (Table 1) nor when grouped by sex (Table 2). The weak genotypic association of rs6465938 (χ^2^ = 6.087, *df* = 2, *p* = 0.048) did not withstand the FDR correction (*p*_FDR_ = 0.713).

**Table 2:** The sex differences in single-SNP association analyses of 30 RELN SNPs in SZ and control cases.

SNPs IDs	Allele						Genotype					
	Man			Woman			Man			Woman		
	χ^2^	df	*p*	χ2	df	*p*	χ^2^	df	*p*	χ2	df	*p*
rs10435342	0.182	1	0.67	0.347	1	0.556	0.564	2	0.754	0.297	2	0.862
rs11496125	0.109	1	0.741	0.023	1	0.88	0.157	2	0.925	0.357	2	0.836
rs11764507	0.029	1	0.864	0.017	1	0.896	2.351	2	0.309	3.808	2	0.149
rs11976900	0.083	1	0.773	0.006	1	0.936	0.074	2	0.964	0.236	2	0.889
rs123713	2.082	1	0.149	0.128	1	0.721	2.956	2	0.228	4.129	2	0.127
rs123714	2.514	1	0.113	0.128	1	0.721	2.864	2	0.239	4.129	2	0.127
rs12705141	0.128	1	0.72	2.408	1	0.121	0.368	2	0.832	2.158	2	0.34
rs144525	0.38	1	0.538	0.873	1	0.35	2.941	2	0.23	1.585	2	0.453
rs1510846	1.12	1	0.29	0.056	1	0.813	1.247	2	0.536	2.278	2	0.32
rs155333	0.039	1	0.844	0.002	1	0.967	3.367	2	0.186	0.485	2	0.785
rs17157643	2.829	1	0.093	0.111	1	0.739	3.603	2	0.165	0.127	1	0.721
rs2229864	0.004	1	0.951	1.338	1	0.247	0.561	2	0.755	2.543	2	0.28
rs2299334	0.645	1	0.422	0.013	1	0.91	1.073	2	0.585	0.077	2	0.962
rs2299356	0.123	1	0.725	2.312	1	0.128	0.626	2	0.731	5.617	2	0.06
rs2535764	0.8	1	0.371	0.079	1	0.779	3.573	2	0.168	0.295	2	0.863
rs262355	0.1	1	0.752	0.025	1	0.874	4.161	2	0.125	0.367	2	0.832
rs362626	0.465	1	0.495	0.142	1	0.706	0.667	2	0.716	4.031	2	0.133
rs362691	1.339	1	0.247	2.304	1	0.129	1.903	2	0.386	2.812	2	0.245
rs362719	0.728	1	0.393	0.986	1	0.321	1.571	2	0.456	1.025	2	0.599
rs362726	1.641	1	0.2	1.01	1	0.315	3.042	2	0.219	1.534	2	0.464
rs362731	0.834	1	0.361	0.141	1	0.707	2.87	2	0.238	1.35	2	0.509
rs362813	0.955	1	0.329	0.141	1	0.707	2.541	2	0.281	1.35	2	0.509
rs362814	0.094	1	0.759	0	1	0.997	0.197	2	0.906	4.083	2	0.13
rs39339	0.915	1	0.339	0.19	1	0.663	1.342	2	0.511	2.89	2	0.236
rs540058	2.504	1	0.114	0.253	1	0.615	2.722	2	0.256	0.258	1	0.612
rs563264	2.682	1	0.102	0.256	1	0.613	2.871	2	0.238	0.26	1	0.61
rs607755	0.314	1	0.575	0.542	1	0.462	1.12	2	0.571	0.756	2	0.685
rs6465938	0.367	1	0.545	0.193	1	0.66	3.56	2	0.169	3.38	2	0.184
rs661575	2.413	1	0.12	3.339	1	0.068	2.166	2	0.338	3.058	2	0.217
rs727708	0.636	1	0.425	3.321	1	0.068	1.458	2	0.482	4.565	2	0.102

### Haplotype Association Analyses

**FIGURE 3:**
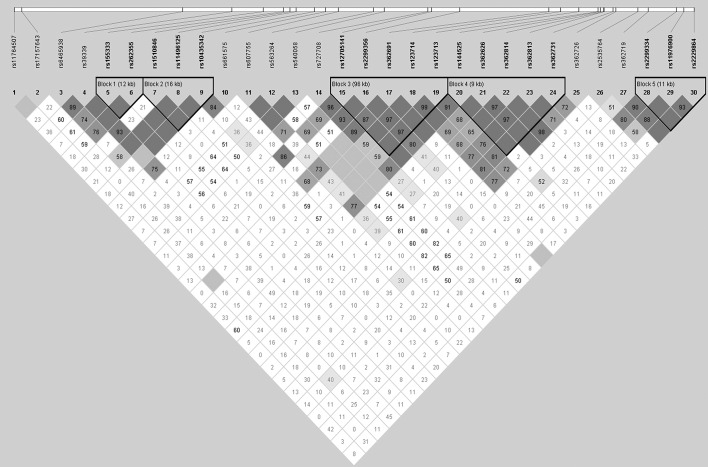
Linkage disequilibrium (LD) among the 30 RELN SNPs in the case and control groups. The pairwise LD *R*^2^ values of the sample set are illustrated in the matrix. The dark color indicates relatively strong LD. The *R*^2^ values of five blocks covering the *RELN* gene were larger than 0.75, indicating reasonable haplotype blocks.

Five blocks were captured using Haploview v4.2 software in the total sample (Figure 3). The frequency and haplotype association within the block were shown in Supplementary Table S1. No haplotype analysis within the block survived before 10000 permutations. Haplotype associations of 2-SNP, 3-SNP, and 4-SNP were analyzed using Unphased v3.1.7 software. When performing logistic regression analyses, only birthplace shown the population stratification [*p* = 0.019, Exp (B) = 0.526, 95% CI 0.308–0.898]. To reduce the stratification, we employed birthplace, sex, and age as covariates in the association analyses. Two 3-SNP haplotype blocks (one consisting of rs362814, rs39339, and rs540058, and the other consisting of rs362691, rs362719, and rs362726) were identified before multiple adjustment (χ^2^ = 19.406, *df* = 6, *p* = 0.004, *p*_Bonf_ = 0.099 and χ^2^ = 20.621, *df* = 7, *p* = 0.004, *p*_Bonf_ = 0.122, respectively). The haplotype with four SNPs (rs362814, rs39339, rs540058, and rs661575) was significantly associated with SZ (χ^2^ = 29.024, *df* = 9, *p* = 6.42E-04), even after Bonferroni and FDR correction (both *p*_adjustment_ = 0.017). And the T-C-T-C haplotype of the four SNPs was more common in the controls (0.764% in case versus 6.173% in controls, OR = 0.050, 95% CI = 0.004–0.705). No sex effect was found in multiple SNP analyses (date not shown).

### QTL Association Analyses

For the QTL analyses, we used smoking, birthplace, age, and sex as covariates, as smoking may be associated with the severity of the negative symptoms (Zhang et al., 2012). Several factors rather than scale score were significantly associated with 3-SNP or 4-SNP haplotypes after FDR correction (Table 3, more information shown in Supplementary Table S2). The T-C-T haplotype and C-T-T haplotype of the 3-SNP consisting of rs2229864, rs2535764 and rs262355 were related to the severity of P7 (hostility) (AddVal = 0.748, 95% CI = 0.291–1.204 and AddVal = 0.718, 95% CI = 0.230–1.206). No significant result was found in SNP or 2-SNP QTL association analyses neither in total sample nor grouped by sex (data not shown). We did not performed 3-SNP and 4-SNP QTL association analyses in male or female patients due to the small sample.

**Table 3:** Positive results of haplotype QTL association analyses.

PANSS item	SNP1 ID	SNP2 ID	SNP3 ID	SNP4 ID	χ^2^	df	*p*	*p*_Bonf_	*p*_FDR_
P7	rs2229864	rs2535764	rs262355		31.424	6	2.10E-05	0.019	0.019
G11	rs12705141	rs144525	rs1510846	rs2299334	36.900	10	5.89E-05	0.052	0.025
G16	rs362814	rs39339	rs540058	rs661575	28.620	7	1.70E-04	0.151	0.025
P7	rs2229864	rs2535764	rs262355	rs362626	37.772	12	1.67E-04	0.149	0.025
G14	rs2229864	rs2535764	rs262355	rs362626	36.621	12	2.57E-04	0.229	0.026
S3	rs2229864	rs2535764	rs262355	rs362626	33.360	11	4.60E-04	0.41	0.041
P7	rs17157643	rs2229864	rs2535764	rs262355	32.060	8	9.09E-05	0.081	0.025
S3	rs17157643	rs2229864	rs2535764	rs262355	29.611	8	2.48E-04	0.221	0.026
G14	rs10435342	rs11496125	rs11764507	rs11976900	33.280	9	1.19E-04	0.106	0.025
P4	rs10435342	rs11496125	rs11764507	rs11976900	25.774	7	5.53E-04	0.493	0.045
P7	rs155333	rs17157643	rs2229864	rs2535764	30.959	8	1.43E-04	0.127	0.025
S3	rs155333	rs17157643	rs2229864	rs2535764	29.445	8	2.65E-04	0.236	0.026

*P4: excitement; P7: hostility; G11: poor attention; G14: Poor impulse control; G16: active social avoidance; S3: emotional instability; p_Bonf_: corrected p-values by Bonferroni correction; p_FDR_: corrected p-values by FDR correction.*

### Power

The G^∗^Power program was used to perform the power calculation. The size of this sample revealed a power of 98.873% to detect a significant association (α < 0.05) when given an effect size index of 0.5 (corresponding to a “medium” gene effect). However, the power narrowed down to 47.033% when the effect size index was 0.2 (corresponding to a “small” gene effect).

## Discussion

Schizophrenia is a complex genetic disease with diverse clinical symptoms. Here, we performed qualitative and quantitative trait association analyses in a Han Chinese population from southern China to verify that *RELN* was a susceptibility gene for SZ. The results of multiple SNP analyses confirmed the genotype-phenotype relationship between *RELN* and SZ.

A 4-SNP haplotype consisting of rs362814, rs39339, rs540058, and rs661575 was observed to be significantly associated with SZ even after the Bonferroni correction, and the T-C-T-C haplotype may be a protective factor for SZ. Moreover, when performing QTL analysis, this 4-SNP haplotype had a significant association with G16 after FDR correction. These results suggested the importance of the four SNPs, which was supported by previous studies. A study of Han population origin from southwestern China demonstrated that the allele of rs362814 was more common in SZ cases and the A and T alleles took part in the building of a risk haplotype and a protective haplotype, respectively (Li et al., 2013). In our study, the T allele of rs362814 also took part in building a protective haplotype in the participants, but with different SNPs. Rs39339 was slightly significant in allelic association tests before the Bonferroni correction and nominally significant in a combined analysis in a Scandinavian population (Kahler et al., 2008). Rs540058 was reported to be associated with the severity of positive symptoms of SZ (Wedenoja et al., 2010), and rs661575 was associated with visual learning and memory in Finnish families (Wedenoja et al., 2008). The four SNPs are intronic and not in high linkage disequilibrium, so they are unlikely to have direct functional impacts on *RELN* and are unlikely to be co-inherited. However, SNPs in different functional areas may affect gene expression in different manners and have different effects on gene function (Manolio, 2010). Moreover, up to 52% of all SNPs that were associated with disease were intronic SNPs (Chen et al., 2010; Manolio, 2010). Hence, further studies on large samples are needed to verify the associations between the four SNPs and SZ, and rs362814 might be associated with SZ in an allele-dependent manner.

Rs2229864 is a synonymous mutation located in the 50th exon of the *RELN* gene. Rs2229864 was recognized to be detrimental in the screening process and was imbalanced in the allelic expression of *RELN* in SZ (Ovadia and Shifman, 2011). Rs2535764 and rs262355 are both intronic SNPs and were slightly significant in the allelic association test only before the Bonferroni correction (Kahler et al., 2008). Both rs262355 and rs2229864 failed to obtain definite positive results in subsequent studies (Li et al., 2011). However, a weak association with P7 and haplotype consisting of rs2229864, rs2535764, and rs262355 withstood both the FDR and Bonferroni correction in the QTL analysis, which demonstrates that the relationship may be genuine rather than a false positive in this study. In addition, 4-SNP haplotypes consisting of the three SNPs and rs362626 or rs17157643 were associated with P7, G14 and S3, or P7 and S3, which all reflect the impulsive and dangerous behavior of SZ patients in PANSS. Both single item scores and scale scores were used as quantitative traits instead of synthesized scale scores in this study to deeply understand the relationships between the severity of clinical symptoms and genetic bases as well as the category of symptoms and genetics. Overall, we presumed that rs2229864, rs2535764, and rs262355 may be closely related to the risk for SZ patients, and subsequent studies with large samples will be especially necessary.

Another interesting phenomenon was that the AA/GG ratio of rs727708 seemingly had opposite trends in the SZ patients and controls, although a significant difference was not found in the allele or genotype association analyses. Both rs727708 and rs540058 were thought to be related to the severity of positive symptoms (Wedenoja et al., 2010), so rs727708 may be related to both the susceptibility to and severity of SZ.

Both rs362691 and rs6465938 failed in single and multiple analyses. Rs362691 is a missense mutation located in the 22th exon of the *RELN* gene and can cause the Leu-Val amino acid change. Rs362691 had also been recognized as a detrimental mutation in the screening process. Researchers have found that rs362691 was not only associated with autism spectrum disorders (ASDs) (Wang et al., 2014) but might also take part in the influence of the *RELN* gene on the cognitive functions of healthy people (Baune et al., 2010). Haplotypes consisting of rs362691 rather than a SNP played roles in susceptibility to SZ in the Chinese Va population (Yang et al., 2013). A subtle but significant difference in the genotype frequency distribution of rs6465938 was found before multiple corrections. Notably, rs6465938 was not in HWE only in the patient samples. Moreover, rs6465938 showed a nominal association in a Scandinavian population (Kahler et al., 2008). Therefore, negative results in this study should not be sufficient evidence to deny the possible associations between rs362691 or rs6465938 and SZ.

When subjects were divided by sex, none of these SNPs was found to be significantly associated with SZ in this study. Due to the small sample size, QTL analyses of different sexes were not performed. Many studies have demonstrated that males and females show different clinical factors, such as age of onset, treatment, cognitive function and clinical symptomatology (Tang et al., 2007; Ochoa et al., 2012; Zhang et al., 2012; Thorup et al., 2014), as well as genetic information (Shifman et al., 2008; Liu et al., 2010). We agree with the notion that male SZ patients are different from female patients in terms of both clinical and genetic conditions. Our negative findings are mainly due to the small sample size and low statistical power, so studies with larger sample sizes would contribute to identifying sex differences in patients with SZ.

There are several possible explanations for the discrepancies between studies, such as genetic heterogeneity in distinct ethnic populations (Caucasian versus East Asian race, Han versus Va populations), the heterogeneity of SZ (subtypes and disease categories), population stratification, environmental exposure, cultures and diets (Li et al., 2011, 2013). Although we tried to reduce the effects of region and SZ subtype, the major limitation of the small sample size due to the rigorous standards still exists. The enrollment of patients principally from an outpatient service instead of an inpatient department may be another reason, as the overall PANSS scores may tend to be lower for outpatients than for hospitalized patients.

## Conclusion

In summary, the current investigation showed qualitative and quantitative trait associations between genetic variants in *RELN* and SZ in a southern Han Chinese population. Several SNPs showed significant associations with the pathogenesis of SZ and/or the severity of psychiatric symptoms principally in multi-SNP analyses. Considering the limitation of our work, further investigations of genetic susceptibility among larger samples and inpatients are required to elucidate the role of the *RELN* polymorphisms in the risk and sex difference of SZ as well as the severity of clinic symptoms in the future.

## Data Availability

The raw data supporting the conclusions of this manuscript will be made available by the authors, without undue reservation, to any qualified researcher.

## Author Contributions

HZ, Y-WS, and J-HC contributed to the conception and design of the study, and provided the approval for publication of the content. XL wrote the protocol and managed the literature searches and SNP screening. SC and LX organized the database and performed the statistical analysis. XL and SC wrote sections of the manuscript. All authors contributed to the manuscript revision and read and approved the submitted version.

## Conflict of Interest Statement

The authors declare that the research was conducted in the absence of any commercial or financial relationships that could be construed as a potential conflict of interest.

## Footnotes

1 https://www.r-project.org/

## Abbreviations

DIGS: the Diagnostic Interview for Genetic Studies
DSM-IV: the Diagnostic and Statistical Manual of Mental Disorders, Fourth Edition
FASTSNP: function analysis and selection tool for single nucleotide polymorphisms
FDR: false discovery rate
FIGS: the Familial Interview for Genetic Studies
GWAS: genome wide association study
HWE: Hardy-Weinberg equilibrium
MAF: minor allele frequency
OMIM: Online Mendelian Inheritance in Man
OR: odds ratios
PANSS: the positive and negative symptoms scale
QTL: quantitative trait loci
SIFT: Sorting Tolerant From Intolerant
SNPs: single nucleotide polymorphisms
SZ: schizophrenia
UTR: untranslated region.

## References

[B1] Adzhubei I. A. Schmidt S. Peshkin L. Ramensky V. E. Gerasimova A. Bork P. (2010). A method and server for predicting damaging missense mutations. *Nat. Methods* 7 248–249. 10.1038/nmeth0410-248 20354512 PMC2855889

[B2] Barrett J. C. Fry B. Maller J. Daly M. J. (2005). Haploview: analysis and visualization of LD and haplotype maps. *Bioinformatics* 21 263–265. 10.1093/bioinformatics/bth457 15297300

[B3] Baune B. T. Konrad C. Suslow T. Domschke K. Birosova E. Sehlmeyer C. (2010). The reelin (RELN) gene is associated with executive function in healthy individuals. *Neurobiol. Learn. Mem.* 94 446–451. 10.1016/j.nlm.2010.08.002 20727978

[B4] Ben-David E. Shifman S. International Schizophrenia Consortium (2010). Further investigation of the association between rs7341475 and rs17746501 and schizophrenia. *Am. J. Med. Genet. B Neuropsychiatr. Genet.* 153B, 1244–1247. 10.1002/ajmg.b.31093 20468075

[B5] Bocharova A. V. Stepanov V. A. Marusin A. V. Kharkov V. N. Vagaitseva K. V. Fedorenko O. Y. (2017). Association study of genetic markers of schizophrenia and its cognitive endophenotypes. *Russ. J. Genet.* 53 139–146. 10.1134/S1022795417010033 29372809

[B6] Chen R. Davydov E. V. Sirota M. Butte A. J. (2010). Non-synonymous and synonymous coding SNPs show similar likelihood and effect size of human disease association. *PLoS One* 5:e13574. 10.1371/journal.pone.0013574 21042586 PMC2962641

[B7] Doss G. P. Rajasekaran R. Arjun P. Sethumadhavan R. (2010). Prioritization of candidate SNPs in colon cancer using bioinformatics tools: an alternative approach for a cancer biologist. *Interdiscip. Sci.* 2 320–346. 10.1007/s12539-010-0003-3 21153778

[B8] Dudbridge F. (2008). Likelihood-based association analysis for nuclear families and unrelated subjects with missing genotype data. *Hum. Hered.* 66 87–98. 10.1159/000119108 18382088 PMC2386559

[B9] Esan O. B. Ojagbemi A. Gureje O. (2012). Epidemiology of schizophrenia–an update with a focus on developing countries. *Int. Rev. Psychiatry* 24 387–392. 10.3109/09540261.2012.725219 23057975

[B10] Falkai P. Rossner M. J. Schulze T. G. Hasan A. Brzozka M. M. Malchow B. (2015). Kraepelin revisited: schizophrenia from degeneration to failed regeneration. *Mol. Psychiatry* 20 671–676. 10.1038/mp.2015.35 25824303

[B11] Faul F. Erdfelder E. Buchner A. Lang A. G. (2009). Statistical power analyses using G^∗^Power 3.1: tests for correlation and regression analyses. *Behav. Res. Methods* 41 1149–1160. 10.3758/BRM.41.4.1149 19897823

[B12] Gabriel S. B. Schaffner S. F. Nguyen H. Moore J. M. Roy J. Blumenstiel B. (2002). The structure of haplotype blocks in the human genome. *Science* 296 2225–2229. 10.1126/science.1069424 12029063

[B13] Guidotti A. Auta J. Davis J. M. Di-Giorgi-Gerevini V. Dwivedi Y. Grayson D. R. (2000). Decrease in reelin and glutamic acid decarboxylase67 (GAD67) expression in schizophrenia and bipolar disorder: a postmortem brain study. *Arch. Gen. Psychiatry* 57 1061–1069. 10.1001/archpsyc.57.11.1061 11074872

[B14] Hartfuss E. Forster E. Bock H. H. Hack M. A. Leprince P. Luque J. M. (2003). Reelin signaling directly affects radial glia morphology and biochemical maturation. *Development* 130 4597–4609. 10.1242/dev.00654 12925587

[B15] Herz J. Chen Y. (2006). Reelin, lipoprotein receptors and synaptic plasticity. *Nat. Rev. Neurosci.* 7 850–859. 10.1038/nrn2009 17053810

[B16] Hill J. J. Hashimoto T. Lewis D. A. (2006). Molecular mechanisms contributing to dendritic spine alterations in the prefrontal cortex of subjects with schizophrenia. *Mol. Psychiatry* 11 557–566. 10.1038/sj.mp.4001792 16402129

[B17] Huang C. C. D’Arcangelo G. (2008). “The Reelin Gene and Its Functions in Brain Development,” in *Reelin Glycoprotein: Structure, Biology and Roles in Health and Disease*, ed. Fatemi S. H. (New York, NY: Springer Science), 1–14.

[B18] Ikeda S. Sasazuki S. Natsukawa S. Shaura K. Koizumi Y. Kasuga Y. (2008). Screening of 214 single nucleotide polymorphisms in 44 candidate cancer susceptibility genes: a case-control study on gastric and colorectal cancers in the Japanese population. *Am. J. Gastroenterol.* 103 1476–1487. 10.1111/j.1572-0241.2008.01810.x 18510611

[B19] Impagnatiello F. Guidotti A. R. Pesold C. Dwivedi Y. Caruncho H. Pisu M. G. (1998). A decrease of reelin expression as a putative vulnerability factor in schizophrenia. *Proc. Natl. Acad. Sci. U.S.A.* 95 15718–15723. 10.1073/pnas.95.26.15718 9861036 PMC28110

[B20] Kahler A. K. Djurovic S. Kulle B. Jonsson E. G. Agartz I. Hall H. (2008). Association analysis of schizophrenia on 18 genes involved in neuronal migration: MDGA1 as a new susceptibility gene. *Am. J. Med. Genet. B Neuropsychiatr. Genet.* 147B, 1089–1100. 10.1002/ajmg.b.30726 18384059

[B21] Kay S. R. Fiszbein A. Opler L. A. (1987). The positive and negative syndrome scale (PANSS) for schizophrenia. *Schizophr. Bull.* 13 261–276. 10.1093/schbul/13.2.261 3616518

[B22] Kuang W. J. Sun R. F. Zhu Y. S. Li S. B. (2011). A new single-nucleotide mutation (rs362719) of the reelin (RELN) gene associated with schizophrenia in female Chinese Han. *Genet. Mol. Res.* 10 1650–1658. 10.4238/vol10-3gmr1343 21863557

[B23] Kumar P. Henikoff S. Ng P. C. (2009). Predicting the effects of coding non-synonymous variants on protein function using the SIFT algorithm. *Nat. Protoc.* 4 1073–1081. 10.1038/nprot.2009.86 19561590

[B24] Li M. Luo X. J. Xiao X. Shi L. Liu X. Y. Yin L. D. (2013). Analysis of common genetic variants identifies RELN as a risk gene for schizophrenia in Chinese population. *World J. Biol. Psychiatry* 14 91–99. 10.3109/15622975.2011.587891 21745129

[B25] Li W. Song X. Zhang H. Yang Y. Jiang C. Xiao B. (2011). Association study of RELN polymorphisms with schizophrenia in Han Chinese population. *Prog. Neuropsychopharmacol. Biol. Psychiatry* 35 1505–1511. 10.1016/j.pnpbp.2011.04.007 21549172

[B26] Lichtenstein P. Yip B. H. Bjork C. Pawitan Y. Cannon T. D. Sullivan P. F. (2009). Common genetic determinants of schizophrenia and bipolar disorder in swedish families: a population-based study. *Lancet* 373 234–239. 10.1016/S0140-6736(09)60072-6 19150704 PMC3879718

[B27] Liu Y. Chen P. L. McGrath J. Wolyniec P. Fallin D. Nestadt G. (2010). Replication of an association of a common variant in the Reelin gene (RELN) with schizophrenia in Ashkenazi Jewish women. *Psychiatr. Genet.* 20 184–186. 10.1097/YPG.0b013e32833a220b 20431428 PMC2901865

[B28] Manolio T. A. (2010). Genomewide association studies and assessment of the risk of disease. *N. Engl. J. Med.* 363 166–176. 10.1056/NEJMra0905980 20647212

[B29] Meyer L. R. Zweig A. S. Hinrichs A. S. Karolchik D. Kuhn R. M. Wong M. (2013). The UCSC genome browser database: extensions and updates 2013. *Nucleic Acids Res.* 41 D64–D69. 10.1093/nar/gks1048 23155063 PMC3531082

[B30] Nabil Fikri R. M. Norlelawati A. T. Nour El-Huda A. R. Hanisah M. N. Kartini A. Norsidah K. (2017). Reelin (RELN) DNA methylation in the peripheral blood of schizophrenia. *J. Psychiatr. Res.* 88 28–37. 10.1016/j.jpsychires.2016.12.020 28086126

[B31] Need A. C. Ge D. Weale M. E. Maia J. Feng S. Heinzen E. L. (2009). A genome-wide investigation of SNPs and CNVs in schizophrenia. *PLoS Genet.* 5:e1000373. 10.1371/journal.pgen.1000373 19197363 PMC2631150

[B32] Niu S. Yabut O. D’Arcangelo G. (2008). The Reelin signaling pathway promotes dendritic spine development in hippocampal neurons. *J. Neurosci.* 28 10339–10348. 10.1523/JNEUROSCI.1917-08.2008 18842893 PMC2572775

[B33] Ochoa S. Usall J. Cobo J. Labad X. Kulkarni J. (2012). Gender differences in schizophrenia and first-episode psychosis: a comprehensive literature review. *Schizophr. Res. Treatment.* 2012:916198. 10.1155/2012/916198 22966451 PMC3420456

[B34] Ovadia G. Shifman S. (2011). The genetic variation of RELN expression in schizophrenia and bipolar disorder. *PLoS One* 6:e19955. 10.1371/journal.pone.0019955 21603580 PMC3095646

[B35] Qiu S. Korwek K. M. Pratt-Davis A. R. Peters M. Bergman M. Y. Weeber E. J. (2006). Cognitive disruption and altered hippocampus synaptic function in reelin haploinsufficient mice. *Neurobiol. Learn. Mem.* 85 228–242. 10.1016/j.nlm.2005.11.001 16376115

[B36] Shen J. Deininger P. L. Zhao H. (2006). Applications of computational algorithm tools to identify functional SNPs in cytokine genes. *Cytokine* 35 62–66. 10.1016/j.cyto.2006.07.008 16919468

[B37] Shifman S. Johannesson M. Bronstein M. Chen S. X. Collier D. A. Craddock N. J. (2008). Genome-wide association identifies a common variant in the reelin gene that increases the risk of schizophrenia only in women. *PLoS Genet.* 4:e28. 10.1371/journal.pgen.0040028 18282107 PMC2242812

[B38] Si T. Yang J. Shu L. Wang X. Kong Q. Zhou M. (2004). The reliability,validity of PANSS and its Implication. *Chin. Ment. Health J.* 1 45–47.

[B39] Tang Y. L. Gillespie C. F. Epstein M. P. Mao P. X. Jiang F. Chen Q. (2007). Gender differences in 542 Chinese inpatients with schizophrenia. *Schizophr. Res.* 97 88–96. 10.1016/j.schres.2007.05.025 17628430

[B40] Thorup A. Albert N. Bertelsen M. Petersen L. Jeppesen P. Le Quack P. (2014). Gender differences in first-episode psychosis at 5-year follow-up–two different courses of disease? Results from the OPUS study at 5-year follow-up. *Eur. Psychiatry* 29 44–51. 10.1016/j.eurpsy.2012.11.005 23394824

[B41] Tost H. Weinberger D. R. (2011). RELN rs7341475 and schizophrenia risk: confusing, yet somehow intriguing. *Biol. Psychiatry* 69:e19. 10.1016/j.biopsych.2010.10.022 21532921 PMC3082769

[B42] Tueting P. Doueiri M. S. Guidotti A. Davis J. M. Costa E. (2006). Reelin down-regulation in mice and psychosis endophenotypes. *Neurosci. Biobehav. Rev.* 30 1065–1077. 10.1016/j.neubiorev.2006.04.001 16769115

[B43] Wang Z. Hong Y. Zou L. Zhong R. Zhu B. Shen N. (2014). Reelin gene variants and risk of autism spectrum disorders: an integrated meta-analysis. *Am. J. Med. Genet. B Neuropsychiatr. Genet.* 165B, 192–200. 10.1002/ajmg.b.32222 24453138

[B44] Wedenoja J. Loukola A. Tuulio-Henriksson A. Paunio T. Ekelund J. Silander K. (2008). Replication of linkage on chromosome 7q22 and association of the regional Reelin gene with working memory in schizophrenia families. *Mol. Psychiatry* 13 673–684. 10.1038/sj.mp.4002047 17684500

[B45] Wedenoja J. Tuulio-Henriksson A. Suvisaari J. Loukola A. Paunio T. Partonen T. (2010). Replication of association between working memory and Reelin, a potential modifier gene in schizophrenia. *Biol. Psychiatry* 67 983–991. 10.1016/j.biopsych.2009.09.026 19922905 PMC3083525

[B46] Wu J. Jiang R. (2013). Prediction of deleterious nonsynonymous single-nucleotide polymorphism for human diseases. *Sci. World J.* 2013:675851. 10.1155/2013/675851 23431257 PMC3572689

[B47] Xu Z. Kaplan N. L. Taylor J. A. (2007). Tag SNP selection for candidate gene association studies using HapMap and gene resequencing data. *Eur. J. Hum. Genet.* 15 1063–1070. 10.1038/sj.ejhg.5201875 17568388

[B48] Yang X. B. Kang C. Liu H. Yang J. (2013). Association study of the reelin (RELN) gene with Chinese Va schizophrenia. *Psychiatr. Gene.* 23:138. 10.1097/YPG.0b013e32835d705c 23277132

[B49] Yuan H. Y. Chiou J. J. Tseng W. H. Liu C. H. Liu C. K. Lin Y. J. (2006). FASTSNP: an always up-to-date and extendable service for SNP function analysis and prioritization. *Nucleic Acids Res.* 34 W635–W641. 10.1093/nar/gkl236 16845089 PMC1538865

[B50] Zhang X. Y. Chen D. C. Xiu M. H. Haile C. N. Sun H. Lu L. (2012). Cigarette smoking and cognitive function in Chinese male schizophrenia: a case-control study. *PLoS One* 7:e36563. 10.1371/journal.pone.0036563 22570726 PMC3343009

[B51] Zhou Z. Hu Z. Zhang L. Liu H. Liu Z. Du J. (2016). Identification of RELN variation p.Thr3192Ser in a Chinese family with schizophrenia. *Sci. Rep.* 6:24327. 10.1038/srep24327 27071546 PMC4829830

