# Supplementary material for: SNP Variation of RELN Gene and Schizophrenia in a Chinese Population: A Hospital-Based Case–Control Study
Source: Front Genet. 2019 Mar 5;10:175. doi: 10.3389/fgene.2019.00175 (PMC6413413; doi:10.3389/fgene.2019.00175)
Supplement: Supplementary file 1 [file Table_1.DOCX]

Supplementary Material

SNP Variation of RELN Gene and Schizophrenia in a Chinese Population: A Hospital-based Case-control Study

**Supplementary Table 1.** **The frequency and association analyses of haplotypes within the blocks in total sample**.

| Block | Haplotype | Freq^a^ | Case Freq^b^. | Control Freq^c^ | χ^2^ | *p^d^* |
| --- | --- | --- | --- | --- | --- | --- |
| Block 1 | GT | 0.706 | 0.710 | 0.704 | 0.024 | 0.878 |
|  | AA | 0.288 | 0.285 | 0.290 | 0.016 | 0.898 |
| Block 2 | CCC | 0.456 | 0.434 | 0.469 | 0.638 | 0.424 |
|  | TTC | 0.313 | 0.310 | 0.315 | 0.014 | 0.904 |
|  | TCC | 0.159 | 0.172 | 0.151 | 0.377 | 0.539 |
|  | TTT | 0.072 | 0.085 | 0.064 | 0.784 | 0.376 |
| Block 3 | AAGCC | 0.305 | 0.306 | 0.305 | 0.001 | 0.979 |
|  | AAGTT | 0.261 | 0.299 | 0.238 | 2.363 | 0.124 |
|  | AGGCC | 0.249 | 0.239 | 0.255 | 0.174 | 0.677 |
|  | TGCCC | 0.104 | 0.075 | 0.121 | 2.896 | 0.089 |
|  | TGGCC | 0.060 | 0.070 | 0.054 | 0.604 | 0.437 |
|  | AGCCC | 0.012 | 0.010 | 0.013 | 0.112 | 0.738 |
| Block 4 | AATTT | 0.361 | 0.360 | 0.361 | 0.001 | 0.981 |
|  | ACACC | 0.296 | 0.338 | 0.270 | 2.796 | 0.095 |
|  | GCACC | 0.182 | 0.152 | 0.200 | 1.910 | 0.167 |
|  | ACATT | 0.149 | 0.130 | 0.160 | 0.907 | 0.341 |
| Block 5 | GAC | 0.355 | 0.391 | 0.333 | 1.882 | 0.170 |
|  | GGC | 0.255 | 0.254 | 0.256 | 0.004 | 0.953 |
|  | AAC | 0.194 | 0.180 | 0.202 | 0.400 | 0.527 |
|  | GAT | 0.194 | 0.174 | 0.205 | 0.762 | 0.383 |

# *^a^ Frequency of haplotype in total sample. ^b^ Frequency of haplotype in patients. ^c^ Frequency of haplotype in controls. ^d^ Uncorrected p values of haplotype association analysis within block.*
