# Supplementary material for: SNP Variation of RELN Gene and Schizophrenia in a Chinese Population: A Hospital-Based Case–Control Study
Source: Front Genet. 2019 Mar 5;10:175. doi: 10.3389/fgene.2019.00175 (PMC6413413; doi:10.3389/fgene.2019.00175)
Supplement: Supplementary file 2 [file Table_2.DOCX]

Supplementary Material

SNP Variation of RELN Gene and Schizophrenia in a Chinese Population: A Hospital-based Case-control Study

**Supplementary Table 2.** **Positive results of haplotype QTL association analyses with factors from PANSS and estimates of confounder effects .**

| PANSS item | SNP IDs | Haplotype | Count | Freq | AddVal | 95%Lo | 95%Hi | haplotype | | | | | confounder | | |
| --- | --- | --- | --- | --- | --- | --- | --- | --- | --- | --- | --- | --- | --- | --- | --- |
|  |  |  |  |  |  |  |  | χ*^2^* | df | p | p_Bonf_ | p_FDR_ | χ*^2^* | df | p ^a, b,c^ |
| **P7** | **rs2229864-rs2535764-rs262355** | C-C-A | 31.210 | 0.159 | 0.000 | 0.000 | 0.000 | 31.424 | 6 | 2.100E-05 | 0.019 | 0.019 | 8.905 | 6 | 0.179^a^ |
|  |  | C-C-T | 95.910 | 0.489 | 0.019 | -0.317 | 0.355 |  |  |  |  |  | 7.447 | 6 | 0.282 ^b^ |
|  |  | C-T-T | 20.870 | 0.107 | 0.718 | 0.230 | 1.206 |  |  |  |  |  | 8.732 | 6 | 0.190 ^c^ |
|  |  | T-C-T | 20.100 | 0.103 | 0.748 | 0.291 | 1.204 |  |  |  |  |  |  |  |  |
| G11 | rs12705141-rs144525-rs1510846 | A-A-C-A | 4.700 | 0.025 | 0.000 | 0.000 | 0.000 | 36.900 | 10 | 5.890E-05 | 0.052 | 0.025 | 2.044 | 10 | 0.996 ^a^ |
|  |  | A-A-C-G | 44.810 | 0.238 | 35.320 | 3.530 | 67.100 |  |  |  |  |  | 23.900 | 10 | 0.008 ^b^ |
|  |  | A-A-T-G | 70.140 | 0.373 | 36.040 | 4.254 | 67.820 |  |  |  |  |  | 33.030 | 10 | 0.000 |
| G16 | rs362814-rs39339-rs540058-rs661575 | A-A-C-C | 5.000 | 0.026 | 0.000 | 0.000 | 0.000 | 28.620 | 7 | 1.700E-04 | 0.151 | 0.025 | 1.608 | 7 | 0.978 ^a^ |
|  |  | A-A-T-C | 72.600 | 0.378 | 0.947 | -0.050 | 1.944 |  |  |  |  |  | 3.398 | 7 | 0.846 ^b^ |
|  |  | A-A-T-T | 24.390 | 0.127 | 0.927 | -0.098 | 1.952 |  |  |  |  |  | 0.964 | 7 | 0.995 ^c^ |
|  |  | T-A-T-C | 36.150 | 0.188 | 1.003 | -0.009 | 2.015 |  |  |  |  |  |  |  |  |
|  |  | T-A-T-T | 31.870 | 0.166 | 0.430 | -0.596 | 1.457 |  |  |  |  |  |  |  |  |
|  |  | T-C-T-C | 1.982 | 0.010 | 49.910 | -169.500 | 269.400 |  |  |  |  |  |  |  |  |
| P7 | rs2229864-rs2535764-rs262355-rs362626 | C-C-A-A | 9.048 | 0.046 | 0.000 | 0.000 | 0.000 | 37.772 | 12 | 1.670E-04 | 0.149 | 0.025 | 6.256 | 12 | 0.903 ^a^ |
|  |  | C-C-A-C | 23.460 | 0.120 | 0.405 | -0.340 | 1.149 |  |  |  |  |  | 3.714 | 12 | 0.988 ^b^ |
|  |  | C-C-T-A | 33.970 | 0.173 | 0.565 | -0.139 | 1.269 |  |  |  |  |  | 5.080E+06 | 12 | 0.000 ^c^ |
|  |  | C-C-T-C | 63.150 | 0.322 | 0.307 | -0.331 | 0.944 |  |  |  |  |  |  |  |  |
|  |  | T-T-A-C | 2.975 | 0.015 | -0.252 | -1.467 | 0.963 |  |  |  |  |  |  |  |  |
|  |  | T-T-T-A | 2.647 | 0.014 | -0.122 | -1.645 | 1.401 |  |  |  |  |  |  |  |  |
| G14 | rs2229864-rs2535764-rs262355-rs362626 | C-C-A-A | 8.167 | 0.042 | 0.000 | 0.000 | 0.000 | 36.621 | 12 | 2.570E-04 | 0.229 | 0.026 | 15.630 | 12 | 0.209 ^a^ |
|  |  | C-C-A-C | 25.800 | 0.132 | 12.800 | -111.000 | 136.600 |  |  |  |  |  | 7.480 | 12 | 0.824 ^b^ |
|  |  | C-C-T-A | 31.760 | 0.162 | 13.070 | -110.700 | 136.900 |  |  |  |  |  | 8.003 | 12 | 0.785 |
|  |  | C-C-T-C | 64.080 | 0.327 | 12.760 | -111.100 | 136.600 |  |  |  |  |  |  |  |  |
| S3 | rs2229864-rs2535764-rs262355-rs362626 | C-C-A-A | 9.230 | 0.048 | 0.000 | 0.000 | 0.000 | 33.360 | 11 | 4.600E-04 | 0.410 | 0.041 | 17.690 | 11 | 0.089 ^a^ |
|  |  | C-C-A-C | 23.610 | 0.122 | 1.446 | -0.123 | 3.016 |  |  |  |  |  | 2.743 | 11 | 0.994 ^b^ |
|  |  | C-C-T-A | 33.510 | 0.173 | 1.680 | 0.141 | 3.219 |  |  |  |  |  | 7.968 | 11 | 0.716 |
|  |  | C-C-T-C | 62.490 | 0.322 | 1.480 | -0.022 | 2.983 |  |  |  |  |  |  |  |  |
| P7 | rs17157643-rs2229864-rs2535764-rs262355 | A-C-C-T | 17.000 | 0.088 | 0.000 | 0.000 | 0.000 | 32.060 | 8 | 9.090E-05 | 0.081 | 0.025 | 8.160 | 8 | 0.418 ^a^ |
|  |  | T-C-C-A | 30.530 | 0.157 | -0.222 | -0.679 | 0.236 |  |  |  |  |  | 8.768 | 8 | 0.362 ^b^ |
|  |  | T-C-C-T | 78.810 | 0.406 | -0.220 | -0.605 | 0.165 |  |  |  |  |  | 3.428E+20 | 8 | 0.000 ^c^ |
|  |  | T-T-C-T | 21.090 | 0.109 | 0.476 | -0.002 | 0.954 |  |  |  |  |  |  |  |  |
| S3 | rs17157643-rs2229864-rs2535764-rs262355 | A-C-C-T | 17.000 | 0.087 | 0.000 | 0.000 | 0.000 | 29.611 | 8 | 2.480E-04 | 0.221 | 0.026 | 9.008 | 8 | 0.342 ^a^ |
|  |  | T-C-C-A | 31.540 | 0.161 | -0.253 | -0.823 | 0.317 |  |  |  |  |  | 6.540 | 8 | 0.587 ^b^ |
|  |  | T-C-C-T | 77.460 | 0.395 | 0.096 | -0.378 | 0.570 |  |  |  |  |  | 2.050 | 8 | 0.979 |
|  |  | T-T-C-T | 21.140 | 0.108 | 0.663 | 0.096 | 1.230 |  |  |  |  |  |  |  |  |
| G14 | rs10435342-rs11496125-rs11764507-rs11976900 | C-C-A-A | 22.930 | 0.119 | 0.000 | 0.000 | 0.000 | 33.280 | 9 | 1.190E-04 | 0.106 | 0.025 | 8.291 | 9 | 0.505 ^a^ |
|  |  | C-C-G-A | 59.450 | 0.310 | -0.667 | -1.077 | -0.256 |  |  |  |  |  | 3.541 | 9 | 0.939 ^b^ |
|  |  | C-C-G-G | 32.650 | 0.170 | -0.157 | -0.575 | 0.261 |  |  |  |  |  | 2.585 | 9 | 0.979 ^c^ |
|  |  | C-T-G-A | 45.490 | 0.237 | 0.043 | -0.346 | 0.431 |  |  |  |  |  |  |  |  |
| P4 | rs10435342-rs11496125-rs11764507-rs11976900 | C-C-A-A | 19.440 | 0.101 | 0.000 | 0.000 | 0.000 | 25.774 | 7 | 5.530E-04 | 0.493 | 0.045 | 6.853 | 7 | 0.444 ^a^ |
|  |  | C-C-G-A | 65.570 | 0.342 | -0.816 | -1.355 | -0.277 |  |  |  |  |  | 3.752 | 7 | 0.808 ^b^ |
|  |  | C-C-G-G | 28.550 | 0.149 | -0.233 | -0.757 | 0.290 |  |  |  |  |  | 3.342 | 7 | 0.852 ^c^ |
|  |  | C-T-G-A | 38.880 | 0.203 | -0.348 | -0.844 | 0.149 |  |  |  |  |  |  |  |  |
| P7 | rs155333-rs17157643-rs2229864-rs2535764 | A-T-C-C | 32.640 | 0.165 | 0.000 | 0.000 | 0.000 | 30.959 | 8 | 1.430E-04 | 0.127 | 0.025 | 11.050 | 8 | 0.199 ^a^ |
|  |  | G-T-C-C | 78.360 | 0.396 | 0.025 | -0.304 | 0.353 |  |  |  |  |  | 8.552 | 8 | 0.381 ^b^ |
|  |  | G-T-T-C | 21.370 | 0.108 | 0.691 | 0.268 | 1.114 |  |  |  |  |  | 6.065 | 8 | 0.640 ^c^ |
| S3 | rs155333-rs17157643-rs2229864-rs2535764 | A-T-C-C | 32.590 | 0.165 | 0.000 | 0.000 | 0.000 | 29.445 | 8 | 2.650E-04 | 0.236 | 0.026 | 10.940 | 8 | 0.205 ^a^ |
|  |  | G-T-T-C | 21.000 | 0.106 | 0.917 | 0.400 | 1.435 |  |  |  |  |  | 6.904 | 8 | 0.547 ^b^ |
|  |  |  |  |  |  |  |  |  |  |  |  |  | 3.977 | 8 | 0.859 ^c^ |
| *P4: Excitement. P7: Hostility. G11: Poor attention. G14: Poor impulse control. G16: Active social avoidance. S3: Emotional. instability. p_Bonf:_ Corrected p values by Bonferroni correction. p_FDR_: Corrected p values by FDR correction; a confounder effects of age; b confounder effects of somke; c:confounder effects of sex.* | | | | | | | | | | | | | | | |
|  |  |  |  |  |  |  |  |  |  |  |  |  |  |  |  |
